# Root-Specific Expression of a Jacalin Lectin Family Protein Gene Requires a Transposable Element Sequence in the Promoter

**DOI:** 10.3390/genes9110550

**Published:** 2018-11-13

**Authors:** Qiong Wu, Neil A. Smith, Daai Zhang, Changyong Zhou, Ming-Bo Wang

**Affiliations:** 1 Citrus Research Institute, Southwest University, Chongqing 400716, China; Scarlett.Woo@outlook.com; 2 Commonwealth Scientific and Industrial Research Organisation (CSIRO) Agriculture and Food, Canberra, ACT 2601, Australia; neil.smith@csiro.au (N.A.S.); anna.zhang@csiro.au (D.Z.)

**Keywords:** Transposable element, DNA methylation, promoter, root-specific expression, DNA demethylase

## Abstract

Transposable elements (TEs) are widespread in the plant genome and can impact on the expression of neighbouring genes. Our previous studies have identified a number of DNA demethylase-regulated defence-related genes that contain TE sequences in the promoter and show tissue-specific expression in Arabidopsis. In this study we investigated the role of the promoter TE insertions in the root-specific expression of a DNA demethylase-regulated gene, *AT5G38550*, encoding a Jacalin lectin family protein. Using a promoter:GUS fusion reporter gene approach, we first demonstrated that the full-length promoter fragment, carrying four TE sequences, contained the essential regulatory information required for root-specific expression and DNA demethylase regulation in Arabidopsis. By successive deletion of the four TE sequences, we showed that one of the four TE insertions, a 201-bp TE fragment of the hAT DNA transposon family, was required for root-specific expression: Deletion of this TE, but not the first two TE sequences, converted the root-specific expression pattern to a constitutive expression pattern in Arabidopsis plants. Our study provides an example indicating an important role of TE insertions in tissue-specific expression of plant defence-related genes.

## 1. Introduction

Transposable elements (TEs) are mobile DNA elements that play a critical role in the genome evolution of eukaryotic organisms. TEs are in general classified into two types, Class I and Class II. Class I elements are retrotransposons (retro-TEs) that transpose through an RNA intermediate using a “copy and paste” mechanism. Class I TEs are the most abundant TEs in plants. Class II TEs are DNA transposons that use DNA intermediates to transpose via the mechanism known as “cut and paste”. TEs comprise a large proportion of the plant genome, ranging from around 18% in Arabidopsis to nearly 85% in maize [1,2]. Apart from changes to genome size, active TE transpositions can change gene and genome structures through transposition, insertion, excision, chromosome breakage, and ectopic recombination. These changes are often associated with changes in gene function or gene expression levels, and can also impact on the phenotypes of plants [3].

While TE insertions into coding regions of genes can result in a loss of gene function or changes in protein sequence, TE insertions into noncoding or gene flanking regions can impact on gene expression levels [1,2]. Recent studies have shown that TE insertions into introns can both enhance or repress gene expression in plants [4]. On the other hand, TE insertions in the upstream flanking region of genes may impact on gene expression by either repressing or enhancing the transcription of genes or conferring spatial and temporal expression patterns. For instance, a study in maize has shown that abiotic stress response genes are enriched for TE insertions in upstream regions within 1 kb of the transcription start site, suggesting that TEs bring stress-responsive *cis*-regulatory elements to the adjacent genes, allowing them to respond to stress conditions [5]. A number of studies have implicated upstream TE insertions in the expression of aluminium tolerance genes [6,7]. The expression of some disease resistance genes are also found to be dependent on the existence of upstream TEs. A TE insertion into the promoter region of a rice blast resistance gene is essential for the expression of the gene and the associated disease resistance [8]. Similarly, the presence of an upstream retro-TE sequence is required for bacterium-responsive expression of a disease resistance gene in Arabidopsis, and this expression pattern is reminiscent of the retro-TE gene itself from which the promoter TE sequence is derived [9].

Transposable elements are the primary target of epigenetic gene silencing mechanisms, including DNA cytosine methylation, histone modification, and small interfering RNA (siRNA)-directed gene silencing. Indeed, the main function of epigenetic gene silencing is to repress TE activity, hence maintaining genome stability. The epigenetic status of TEs can affect the expression of adjacent genes. Studies in Arabidopsis have shown that genes close to silenced TEs tend to have low levels of expression, and that gene expression levels are negatively correlated with the density of nearby TEs associated with methylation or methylation-causing siRNAs [10,11]. Therefore, genes associated with TEs, such as those stress-responsive and disease resistance genes containing upstream TEs, can also be subject to epigenetic regulation conferred by TEs. A recent study in rice has indicated that epigenetic control of regulatory TE sequences contributes to the fine tuning of disease resistance gene expression, giving a balance between defence and yield [12]. 

We have previously reported that a subset of plant defence-related genes are controlled by DNA demethylases in Arabidopsis [13]. These genes are downregulated in the DNA demethylase mutant, *rdd*, that has loss-of-function mutations in the Arabidopsis DNA demethylase genes, *Ros1*/*Demeter-like 1*, *Demeter-like 2*, and *Demeter-like 3*. Importantly, the upstream promoters of these *rdd*-downregulated defence-related genes are highly enriched for TE insertions, and these TE sequences have increased DNA methylation in *rdd* compared to wild-type Arabidopsis. This suggests that the promoter TE sequences control the expression of the defence-related genes, their regulatory activity can be repressed by DNA methylation, and DNA demethylases are required to reduce TE methylation and maintain active expression of the genes. We have also recently shown that these promoter TE-associated genes are upregulated by infection with the fungal pathogen, *Fusarium oxysporum*, and tend to give root or shoot-specific expression patterns [14], suggesting that the TEs may contain Fusarium-responsive and tissue-specific *cis*-regulatory elements.

To understand the role of promoter TE insertions in the tissue-specific expression patterns of the *rdd*-regulated defence-related genes, we dissected the four TE sequences in the promoter of the Jacalin lectin family protein gene (*AT5G38550*) that shows root-specific expression in Arabidopsis. Using the β-glucuronidase (GUS) reporter gene system, we demonstrate that the root specificity of *AT5G38550* expression is dependent on the presence of a hAT DNA transposable element in the promoter.

## 2. Materials and Methods

### 2.1. Preparation of Promoter:GUS Fusion Constructs

The rice ragged stunt oryzavirus segment 4 promoter (Stunt 4 promoter) from the pPLEX vector [15] and the intron-interrupted hygromycin resistance gene sequence encoding hygromycin phosphotransferase (HPT) with the *Agrobacterium tumefaciens* T-DNA-borne tumour morphology large gene terminator (Tml) from pWBVec8 [16] were assembled into pBluescript KS+ (Stratagene, La Jolla, CA, USA) to form a Stunt 4 promoter:Intron-HPT:Tml cassette. This cassette was released using *Kpn*I/*Bam*HI restriction and ligated into the binary vector, pWBVec1, [16] at the same sites to form Vec1-S4:HPT:Tml. The open reading frame (ORF) of the GUS reporter gene fused to the *A. tumefaciens* Ti plasmid-encoded octopine synthase gene (OCS) terminator sequence (GUS: OCS) was inserted into S4:HPT:Tml/Vec1 at the *Not*I and *Apa*I sites to form the universal binary vector (pQW028) comprising LB:GUS:OCST: Stunt 4: Intron-HPT:Tml1:RB, which harbours a unique *Not*I site in front of the GUS ORF for the introduction of different promoter elements.

Promoter fragments of *AT5G38550*, *AT5G39110*, *AT4G04570*, and *AT1G58602*, and the four truncated promoter fragments of *AT5G38550*, were PCR-amplified from *Arabidopsis thaliana* Columbia (Col-0) DNA using the Phusion^®^ High-Fidelity DNA Polymerase (New England Biolabs, Ipswich, MD, USA) with the respected primer pairs listed in Table S1. Polymerase chain reaction (PCR) cycles included 95 °C for 3 min, 33 cycles of 95 °C for 30 s, 56 °C for 45 s, and 72 °C for 1 min, followed by 72 °C for 10 min. PCR product was first cloned into pGEM-T Easy vector (Promega, Madison, WI, USA), and sequenced using a BigDye Terminator v3.1 Cycle Sequencing Kit (Thermo Fisher Scientific, Waltham, MD, USA) to verify the sequences. The promoter fragments were then excised with NotI and cloned into pQW028 to form the promoter:GUS:OCS fusion constructs.

### 2.2. Transformation of Arabidopsis

The promoter:GUS fusion constructs were introduced into *Agrobacterium tumefaciens* GV3101 using triparental mating [17], and resulting single colonies were verified by plasmid DNA extraction using the alkaline lysis method and diagnostic restriction enzyme digestion. For Arabidopsis transformation, the Agrobacterium strains were grown in liquid LB (Luria-Bertani) medium containing rifamipicin (20 mg/L) and spectinomycin (50 mg/L) for 24 h, collected by centrifugation at 5000 rpm for 10 min, and then resuspended in 5% sucrose solution with 0.03% Silwet L77. Wild-type *Arabidopsis thaliana* Col-0 and the triple DNA demethylase gene mutant, *ros1 dml2 dml3* (*rdd*; [13]), were transformed with the Agrobacterium cells using the “floral dip” method [18].

To obtain transgenic plants, seed from Agrobacterium-treated plants were sterilized first for 3 h with chlorine gas (generated by adding 3 mL hydrochloride acid into 100 mL White King Bleach containing 4.2% sodium hypochlorite) in a sealed desiccator and then with Bleach:ethanol (1:2 ratio in volume) solution for 10 min. The seed was washed with ethanol for four times to remove residual Bleach, air-dried, and spread on MS medium containing 20 mg/L hygromycin B and 150 mg/L timentin. The plates were placed in a 4 °C cold room for 48 h and then incubated in a 22 °C growth room with a 16 h/8 h light/dark light regime. Hygromycin resistant transgenic plants were transferred to new MS (Murashige and Skoog) plates and then to soil.

### 2.3. Histochemical and Fluorometric Assay of GUS Expression

Visualization of GUS expression was performed using histochemical staining of Arabidopsis seedlings with 2 mM X-gluc (5-Bromo-4-chloro-3-indolyl-β-d-glucuronide) [19]. Quantitative assay of GUS activity was performed using the kinetic fluorometric 4-methylumbelliferryl β-d-glucuronide (MUG) assay [20].

### 2.4. DNA Methylation Analysis Using Bisulfite Sequencing

DNA was isolated from a pool of 10–20 Arabidopsis plants of each transgenic line using a CTAB (cetyl trimethylammonium bromide) extraction procedure: Plants were ground in liquid nitrogen to a fine powder, which was suspended quickly in 65 °C 1× CTAB buffer [21] and incubated at 65 °C for approximately 20 min. The slurry was then mixed with chloroform and centrifuged for 10 min at 4000 rpm. The supernatant was transferred to a fresh tube and mixed with 0.7 volume of isopropanol. DNA was pelleted by centrifugation for 10 min at 4000 rpm. To purify the DNA, the pellet was dissolved in 0.5 mL Tris-EDTA (TE) buffer, and DNA precipitated with 50 µL of 3 M NaOAC and 1 mL ethanol on ice for 15 min, centrifuged at 4000 rpm for 10 min, washed with 70% ethanol, air-dried, and dissolved in TE buffer.

Approximately 2 µg of DNA was bisulfite-converted using the EpiTect Plus DNA Bisulfite Kit (Qiagen, Germantown, MD, USA) following the manufacturer’s instruction, yielding 50 µL of converted DNA solution. To check the efficiency of the bisulphite conversion, PCR amplification was first performed on a 157-bp sequence of the chloroplast encoded psaA protein gene using the primers 5′ATGATGTTGTTAGAATTTYATATAGG3′ (forward; Y stands for mixed C and T) and 5′CATCATTTARCTATCRCAATTCTTT3′ (reverse; R stands for mixed G and A). All bisulfite PCR reactions were performed using the following PCR cycles: 12 min at 94 °C followed by 10 cycles of 1 min at 94 °C, 2:30 min at 50 °C, 1:30 min at 72 °C, and 30 cycles with 1 min at 94 °C, 1:30 min at 55 °C, 1:30 min at 72 °C, with a final extension of 10 min at 72 °C. PCR product was directly sequenced to check for conversion of cytosines to thymines [22].

To amplify the promoter sequences of the promoter:GUS fusion transgenes from the bisulfite-converted DNA, nested PCR was employed, as described in Finn et al. [22]. The sequences of the primary and nested PCR primers are listed in Table S1. Nested PCR product was purified using a Qiagen PCR purification kit and directly sequenced using one of the nested primers [22]. To determine DNA cytosine methylation levels, trace file data of the sequenced PCR products were opened using the BioEdit software (http://www.mbio.ncsu.edu/bioedit/bioedit.html), exported to Microsoft Excel using the “Export trace values (tabdelimited text)” feature, and the relative peak heights of cytosines and thymines calculated to indicate the relative degree of methylation at each cytosine location [13].

## 3. Results

### 3.1. Promoter:GUS Fusion Transgenes Show Repressed Expression in rdd Reminiscent of the Corresponding Endogenous Defence-Related Genes

To investigate the role of promoter TEs in the regulation of *rdd*-regulated defence-related genes, we first examined if the promoter fragments with TE sequences contained the necessary information required for the expression pattern of the corresponding endogenous genes using promoter:GUS fusion constructs. We first tested the promoter fragments of four defence-related genes, *AT5G38550* (Jacalin lectin family protein), *AT5G39110* (germin-like protein), *AT4G04570* (cysteine-rich receptor-like kinase), and *AT1G58602* (LRR and NB-ARC domains-containing disease resistance protein). All four genes are (i) strongly downregulated in *rdd*, (ii) upregulated upon Fusarium infection, and (iii) contain TE sequences in the upstream promoter region ([13,14]; Figure S1). In addition, three of them (*AT5G38550*, *AT5G39110*, *AT1G58602*) have also been shown to have root-(*AT5G38550*, *AT5G39110*) or shoot-(*AT1G58602*) specific or preferential expression patterns [14].

**Figure 1:**
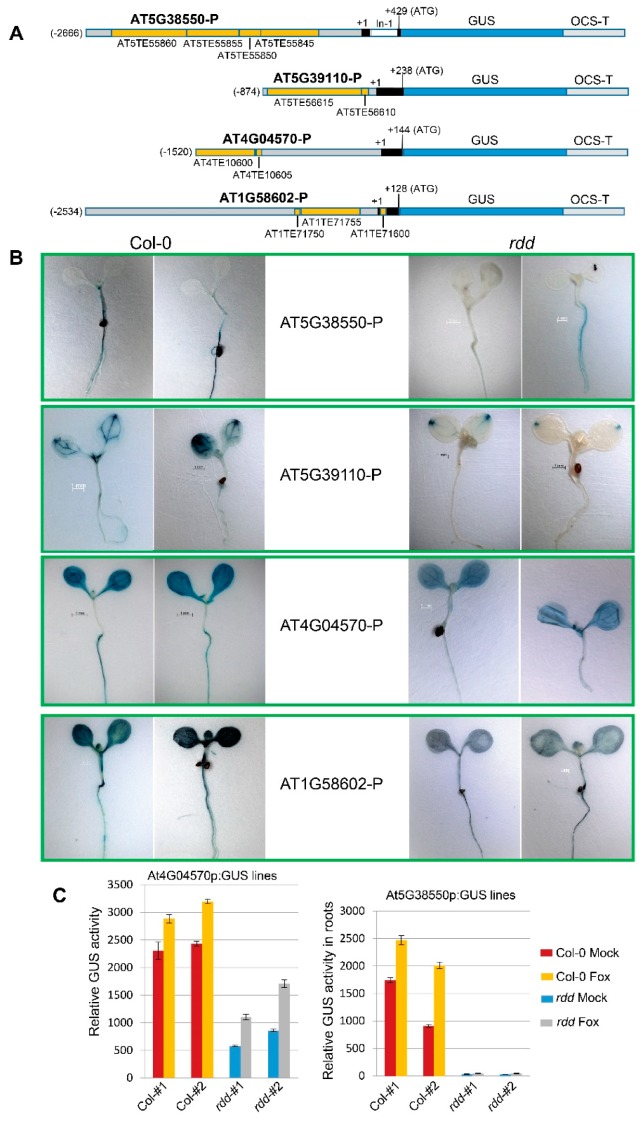
Expression pattern of the promoter:β-glucuronidase (GUS) fusion transgenes in wild-type Col-0 and *rdd* mutant Arabidopsis transgenic lines. (**A**) Schematic diagrams of the promoter:GUS fusion constructs. (**B**) The typical expression patterns of the four promoter:GUS fusion transgenes. (**C**) Fluorometric 4-methylumbelliferryl β-d-glucuronide (MUG) assay of T2 plants from two independent transgenic lines each of the At4G04570p:GUS (whole plants) and At5G38550p:GUS (roots only) constructs in Col-0 or *rdd* backgrounds. GUS expression was analysed at one day post inoculation with *Fusarium oxysporum* (Fox). “+1” in (**A**) indicates the transcription start site of the corresponding genes predicted in TAIR10. All promoter fragments end at the nucleotide immediately before the translational start codon, ATG. GUS, the coding sequence of the β-glucuronidase gene; OCS-T, the transcription terminator sequence of the *Agrobacterium tumefaciens* Ti plasmid-encoded octopine synthase gene.

The promoter fragments are 3.10 kb (AT5G38550-P), 1.11 kb (AT5G39110-P), 1.66 kb (AT4G04570-P), and 2.66 kb (AT1G58602-P) in size, all including the full 5′ UTR (untranslated region) and for AT5G38550-P also including the first intron upstream of the translation start codon, ATG (Figure 1A). All four promoter fragments contain the TE regions that showed differential DNA methylation between *rdd* and wild-type Col-0 plants, hence were implicated in the *rdd* downregulation of the corresponding genes [13,14]. 

The promoter fragments were transcriptionally fused with the GUS coding sequence to generate the promoter:GUS fusion constructs (Figure 1A). In order to transform the *rdd* mutant that was derived from Salk transfer DNA (T-DNA) insertion lines with a kanamycin resistance selectable marker, the constructs were cloned into a binary vector with the hygromycin resistance gene (*HPT*) as the selectable marker. In addition, we used a promoter derived from the genomic segment 4 of rice ragged stunt oryzavirus (Stunt4 promoter) [15] to drive the expression of the *HPT* gene. This was to avoid potential transcriptional cosuppression by the existing sequences in the T-DNA insertions of the *rdd* mutant. The regularly used CaMV 35S promoter is often silenced when introduced into the Salk T-DNA mutant background due to sequence-specific cosuppression by the T-DNA insertion [23].

The constructs were transformed into both Col-0 and *rdd*, resulting in 12 to 28 independent primary (T1) transgenic lines (Table S2). T2 progeny of these lines were analyzed for GUS expression pattern using 5-bromo-4-chloro-3-indolyl glucuronide (X-Gluc) staining, and representative staining patterns of two independent lines for each construct in Col-0 and *rdd* backgrounds are shown in Figure 1B. At1G58602p:GUS lines displayed the most intense, constitutive, GUS staining among the four groups of transgenic lines, followed by the At4G04570p:GUS lines, which also showed constitutive GUS staining in leaf and root tissues (Figure 1B). This is consistent with the relative expression levels of the four corresponding genes determined by microarray analysis (Figure S1). For all four promoter constructs, the intensity of GUS staining was lower in *rdd* than in Col-0, suggesting that the promoters in the transgenes are regulated by the DNA demethylases the same way as in the corresponding endogenous genes.

Quantitative fluorometric 4-methylumbelliferyl β-D-glucuronide (MUG) assay of the At4G04570p:GUS and At5G38550p:GUS transgenic lines confirmed the repressed expression in *rdd* (Figure 1C). Furthermore, the GUS expression levels in these lines were up-regulated upon *Fusarium oxysporum* (Fox) infection (Figure 1C). This result indicated that the promoter fragments in the GUS fusion constructs contain the *cis*-regulatory information for DNA demethylase-mediated regulation as well as for Fusarium-inducible expression of the corresponding defence-related genes. 

To further investigate if the transgene promoters are regulated by the DNA demethylases in the same manner as the endogenous gene promoters, we performed bisulfite sequencing analysis on the promoter fragment in the At4G04570p:GUS transgene. Bisulfite treatment of DNA converts unmethylated cytosines (C) to uracils, which appear as thymines (T) in the PCR product, but 5′-methyl cytosines are resistant to bisulfite treatment. This allows the methylated cytosines to be identified at the single nucleotide level. Efficient bisulfite conversion of DNA was verified by PCR amplification and sequencing of a chloroplast gene, *psaA*, that is known to have no DNA methylation (Figure S2) [22].

**Figure 2:**
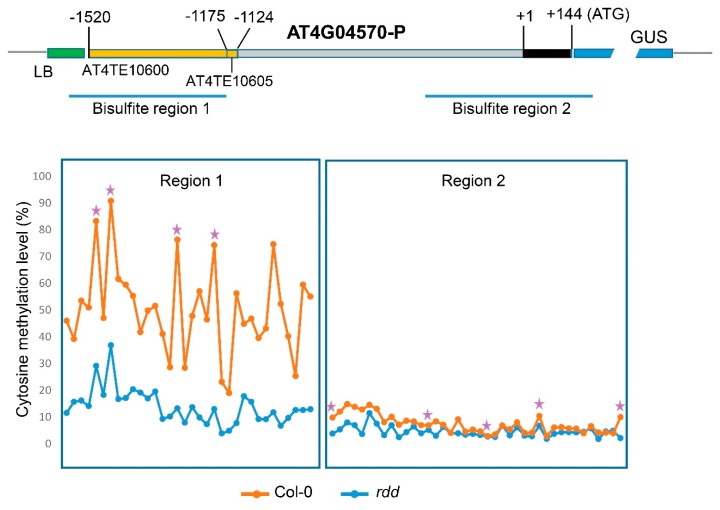
The *AT4G04570* promoter in the GUS fusion transgene shows the same DNA methylation pattern as in the endogenous *AT4G04570* gene. The asterisks indicate the “CG” context.

Our previous study showed that the promoter of the endogenous *AT4G04570* gene has reduced CHH methylation in *rdd* compared to Col-0 in the upstream TE region, but has no DNA methylation in the non-TE sequences near the transcription start site (TSS) [13]. Using transgene-specific primer pairs (T-DNA left border and GUS-specific primers paired with At4G04570p-specific primers) for bisulfite sequencing, we showed that the *AT4G04570* promoter in the GUS fusion transgene had higher levels of CHH (‘H’ stands for A, C or T nucleotide) methylation across the TE region in the Col-0 line than in the *rdd* line, but had no significant DNA methylation in the non-TE regions in both backgrounds (Figure 2). This result indicated that the promoter in the transgene gained a similar DNA methylation pattern as the endogenous gene promoter in both *rdd* and Col-0, and suggested that the differential expression of the At4G04570p:GUS transgene between the *rdd* and Col-0 lines was determined by the differential CHH methylation in the TE region of the promoter. This result also indicated that a loss of DNA demethylase activity in the *rdd* mutant does not result in non-specific DNA methylation in transgene promoters, as almost no promoter DNA methylation was detected in the At4G04570p:GUS transgenic lines of the *rdd* background (Figure 2).

**Figure 3:**
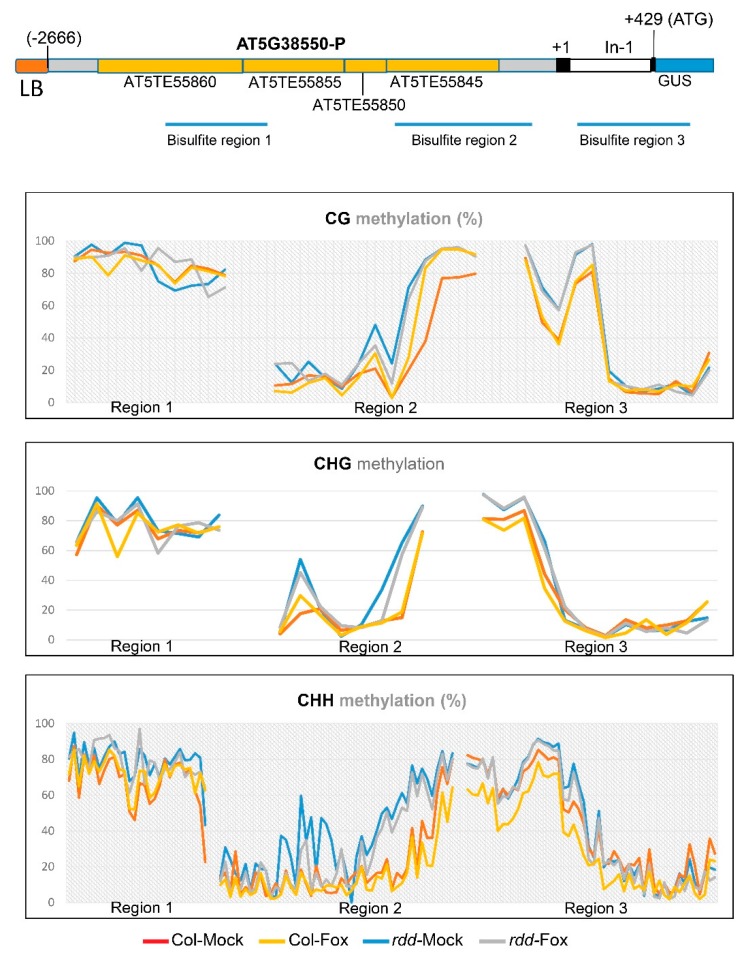
The *AT5G38550* promoter in the GUS fusion transgene shows differential DNA methylation between *rdd* and Col-0 around the TE near the transcription start site.

We also analyzed the DNA methylation status in the *AT5G38550* promoter of the transgenic At5G38550p:GUS plants. Our previous study of the endogenous *AT5G38550* promoter showed strong differential DNA methylation between *rdd* and Col-0 around the TE that is proximal to TSS, with high levels of DNA methylation at all cytosine contexts (CG, CHG, and CHH; H stands for A, C, and T) in *rdd*, but almost no DNA methylation in Col-0 [13]. As shown in Figure 3, differential DNA methylation also occurred in the transgenic promoter, especially around the TE region close to TSS, with higher CG, CHG, and CHH methylation levels in *rdd* than in Col-0. This increase in DNA methylation in *rdd* is consistent with that of the endogenous promoter. However, the transgene promoter has significant levels of DNA methylation near TSS in the Col-0 background, which is different to the endogenous promoter, which had almost no methylation at this region in Col-0 [13]. It is possible that this methylation occurred during plant transformation, which is known to induce de novo DNA methylation to repeat elements in promoters [24]. Nevertheless, the differential DNA methylation of the transgene promoter around the TE near TSS was likely to account for the differential GUS expression between *rdd* and Col-0, as other regions showed less methylation variation. Interestingly, the Fusarium-infected samples showed lower levels of CHH methylation than the mock-treated samples at the differentially methylated region (Figure 3), which coincides with the upregulation of the At5G38550p:GUS transgene in Fusarium infected tissues (Figure 1C). This suggests that the differential CHH methylation contributes to the Fusarium-inducible expression pattern.

### 3.2. The 3.10 kb Promoter Fragment of At5G38550 Contains the *cis*-Regulatory Information Required for Root-Specific Expression

The At5G39110p:GUS lines of the Col-0 background displayed strong GUS expression in the main veins of leaf tissues, particularly in leaf tips (Figure 1B). The transgenic lines in the *rdd* background also showed strong GUS staining in leaf tips, but with only weak GUS staining in leaf veins. Root tissues of the At5G39110p:GUS lines showed relatively weak GUS staining even in the Col-0 background, which was different to the root-preferential expression pattern of the endogenous At5G39110 gene observed previously using RT-PCR (reverse transcription polymerase chain reaction) analysis [14] and reported in the AtGenExpress database based on microarray analysis [25]. The At4G04570p:GUS plants showed relatively uniform GUS staining in both shoots and roots, suggesting that *AT4G04570* is a constitutively expressed gene. No previous report was found on the expression pattern of this gene.

The At1G58602p:GUS lines displayed stronger GUS staining in aerial tissues than root tissues, especially in the Col-0 background, which is consistent with the shoot-preferential expression pattern of the corresponding endogenous gene based on RT-PCR analysis [14].

At5G38550p:GUS lines showed strong root-specific expression of GUS in Col-0, with strong GUS staining in roots, but almost no GUS staining in shoots (Figure 1B), and this staining pattern was highly consistent among independent transgenic lines. The root-specific expression pattern is maintained in the *rdd* transgenic lines, although the level of GUS expression in roots was relatively low (Figure 1B). This GUS expression pattern resembles that of the endogenous *AT5G38550* gene, showing strong root-specific expression as determined using RT-PCR analysis [14] or microarray analysis (AtGenExpress; [25]). These results indicated that the promoter fragments of *At1G58602* and *At5G38550*, especially of the latter, contain the necessary *cis*-regulatory elements required for shoot or root-specific gene expression.

### 3.3. The Root-Specific Expression Pattern of the AT5G38550 Promoter Is Determined by a hAT TE Sequence

**Figure 4:**
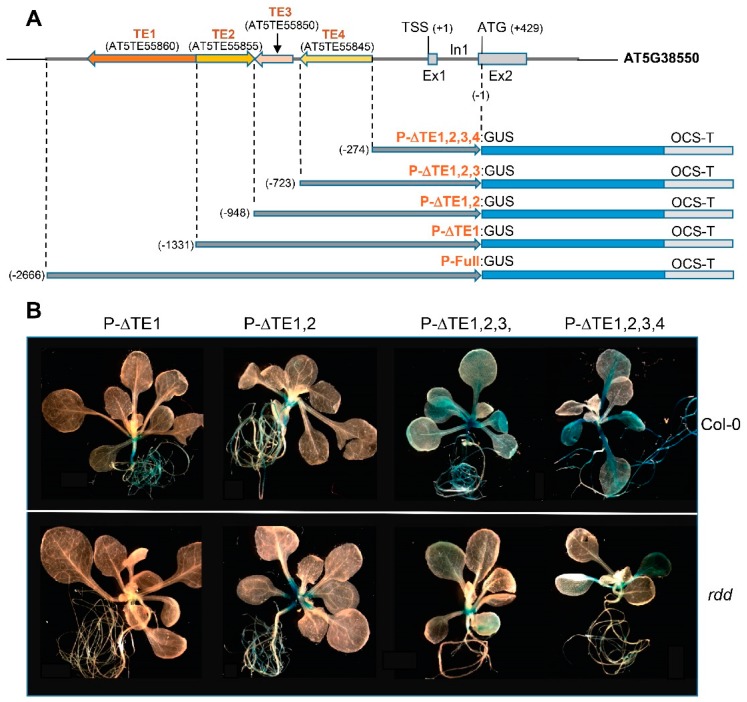

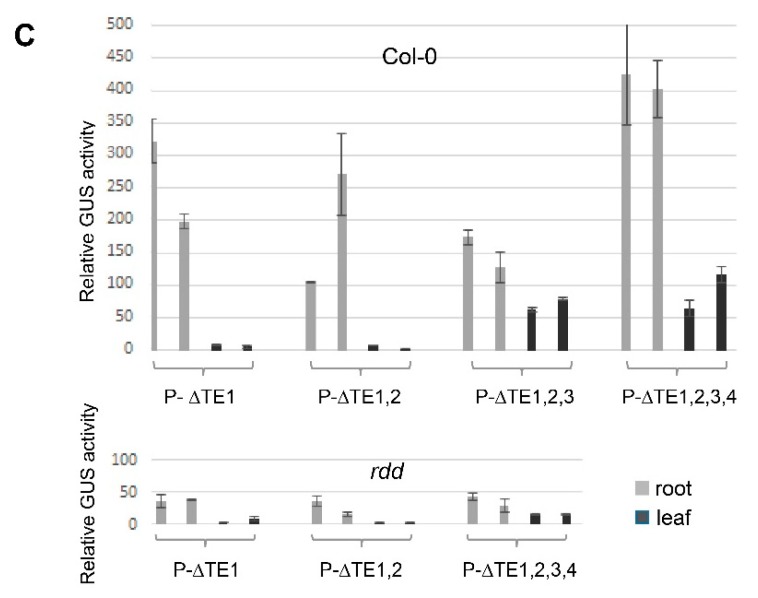
Deletion of a hAT TE sequence abolishes root-specific expression of the *AT5G38550* promoter. (**A**) The structure of the truncated *AT5G38550* promoter:GUS fusion constructs along with the full-length construct P-Full:GUS that is the same as the At5G38550p:GUS shown in Figure 1A. The structure of the *AT5G38550* genomic region is shown above. (**B**,**C**) GUS expression analysis of T3 plants of the truncated *AT5G38550* promoter:GUS lines.

Our previous study suggested that the shoot and root-specific expression patterns of the *rdd*-downregulated defence-related genes are independent of the DNA methylation level in the promoter TEs; only subtle difference in DNA methylation was detected between shoots and roots, and the tissue-specific pattern occurs in both Col-0 and *rdd* despite a reduced level of expression in the latter [14]. Based on this, we proposed that the tissue-specific expression patterns are conferred by *cis*-regulatory sequence elements situated inside the promoter TEs, and that DNA methylation affects the activity, but not the tissue specificity, of the element. To test this, we dissected the *AT5G38550* promoter, which gave the strongest tissue-specific expression pattern among the four promoters. The *AT5G38550* promoter contains sequences of four different TEs, including three DNA transposons hAT (*AT5TE55860*), Helitron (*AT5TE55855*), and hAT (*AT5TE55850*), and a LINE retro-TE (*AT5TE55845*) (Figure 1A). We generated four truncated *AT5G38550* promoter:GUS constructs, in which the four TE fragments were successively deleted (Figure 4A). These constructs were named P-ΔTE1:GUS (with the first TE or TE1 deleted), P-ΔTE1,2:GUS (with the upstream two TEs deleted), P-ΔTE1,2,3:GUS (with three TEs deleted), and P-ΔTE1,2,3,4:GUS (with all four TEs deleted) (Figure 4A). These constructs were then transformed into Col-0 and *rdd*, resulting in 11 to 22 independent transgenic lines (Table S2), and these transgenic plants were analysed for GUS expression.

Histochemical X-gluc staining of T2 transgenic plants in the Col-0 background showed that P-ΔTE1:GUS and P-ΔTE1,2:GUS lines showed the same root-specific expression pattern as the full-length At5G38550p:GUS lines (Figure S3), indicating that deletion of the first two TEs had no effect on the tissue-specific expression pattern. However, this root-specific expression pattern was switched to constitutive GUS expression in the P-ΔTE1,2,3:GUS and P-ΔTE1,2,3,4:GUS lines, where GUS expression was clearly detected in both roots and shoots (Figure S3). Thus, deletion of the third TE sequence, a 201-bp partial sequence of the TAG3N1 DNA TE family in the hAT superfamily, abolished the root-specific expression pattern. This result suggested that the hAT TE sequence, or together with the TE1 and TE2 sequences, is required for the root specificity of the *AT5G38550* promoter.

We obtained T3 plants from the truncated *AT5G38550* promoter:GUS lines, and analysed GUS expression patterns. As shown in Figure 4B, the root-specific expression pattern of the P-ΔTE1:GUS and P-ΔTE1,2:GUS lines in the Col-0 background was retained in the T3 generation. Similarly, like the T2 plants, the T3 plants of the P-ΔTE1,2,3:GUS and P-ΔTE1,2,3,4:GUS lines showed GUS staining in both roots and shoots, indicating the loss of root specificity. Quantitative fluorometric MUG assay confirmed the GUS staining patterns, showing almost no GUS activity in the leaf tissues of T3 P-ΔTE1:GUS and P-ΔTE1,2:GUS plants, but dramatically increased GUS activity in the leaves of the T3 P-ΔTE1,2,3:GUS and P-ΔTE1,2,3,4:GUS plants (Figure 4C). This result confirmed the requirement of the short TE fragment, or together with the TE1 and TE sequences, for the root-specific expression pattern.

Analysis of GUS expression in the truncated promoter:GUS lines of the *rdd* background showed that the truncated promoters continued to be repressed in the *rdd* mutant: GUS activity was dramatically lower in the T3 plants of *rdd* lines than those of the Col-0 background (Figure 4B). This was true for all the four truncated constructs. The repressed GUS expression in *rdd* was confirmed by MUG assay of the T3 P-ΔTE1:GUS, P-ΔTE1,2:GUS, and P-ΔTE1,2,3,4:GUS plants (T3 P-ΔTE1,2,3:GUS plants were not available for MUG assay) (Figure 4C). This repressed GUS expression in the P-ΔTE1,2,3,4:GUS plants suggested that the four TE sequences were not the only elements required for DNA demethylase-mediated regulation of the *AT5G38550* gene, and that the non-TE sequence near TSS is also important. This is consistent with our previous study showing that the differentially methylated region in the *AT5G38550* promoter extends beyond the TE sequence into the non-TE sequence near TSS [13]. However, despite the repressed GUS expression in *rdd*, root-specific expression was retained for the P-ΔTE1:GUS and P-ΔTE1,2:GUS plants of *rdd* background and lost in the P-ΔTE1,2,3:GUS and P-ΔTE1,2,3,4:GUS plants that showed significant GUS expression in leaf tissues (Figure 4B,C). This further indicated that the third TE fragment, the short hAT TE sequence, is required for conferring root-specific expression of the *AT5G38550* gene. 

## 4. Discussion

TE insertions near genes are a common occurrence in plants. It has been reported that 36% of Arabidopsis genes have TEs within 1 kb while this figure is 86% for maize [26]. There is increasing evidence in recent years that these near-gene TE insertions contribute to the expression diversity of plant genes, particularly of those genes involved in environmental stress responses. In animals, TE insertions have recently been recognized as a major source of *cis*-regulatory elements conferring novel gene expression patterns to adjacent genes, such as cell-type specific and species-specific gene expression patterns [27,28]. In plants, TE insertions in upstream promoter regions have been shown in a number of recent studies to be important for the expression of the adjacent genes, such as disease resistance and aluminium tolerance genes [6,7,8,9]. However, little is known about the role of upstream TE insertions in tissue-specific expression of genes. 

In this study, we investigated the involvement of promoter TE insertions in the root-specific expression pattern of a *rdd*-downregulated Arabidopsis defence gene, the jacalin lectin family protein gene, using a promoter:GUS reporter gene approach. The promoter of this gene has four different TE sequences (Figure S4), and by sequentially deleting the TE sequences, we demonstrated that loss of the third TE sequence, a 201 bp partial sequence (*AT5TE55850*) of the TAG3N1 family TE in the hAT DNA transposon superfamily, converted root-specific expression of GUS to constitutive expression in both roots and leaves. This result suggested that the hAT TE fragment contains *cis*-regulatory sequences conferring root-specific expression.

Searching the 201-bp hAT TE sequence against the AthaMAP database [29] identified a number of possible transcription factor-binding sites, such as AHL20, ARR11, and RVE1 binding motifs (Table S3). Two of the transcription factor genes, *AHL20* and *ARR11*, showed root-specific and root-preferential expression patterns, respectively, in the AtGenExpress database (Table S3; [25]) and also in Imamura et al. for *ARR11* [30]. These transcription factors can therefore potentially interact with the AT5G38550 promoter at the hAT sequence to control the root-specific expression patterns. AHL20 is a member of the AT-hook family, which recognises AT-rich sequence motifs, such as AAAT and AATT [31]. There are a number of putative binding sites for AHL20 in the hAT TE sequence, including a relatively long stretch of AT-rich sequence near the 5′ end containing two “AAAT” motifs (Figure S4). The GARB/ARR-B family transcription factor, ARR11, has been reported to be involved in cytokinin and glucose-mediated root growth and development in Arabidopsis [32]. The optimal sequence motif for ARR11, “GGATT” [30], occurs twice in the hAT TE, but only once elsewhere in the whole promoter sequence (Figure S4). This is in contrast to AHL20-binding motifs that occur many times across the whole promoter sequence, making the ARR11 motifs a more likely candidate for the root-specific *cis*-element of the *AT5G38550* promoter. However, more detailed dissection is needed to determine the exact sequence element in the hAT sequences for root-specific expression.

The jacalin lectin family protein gene, *AT5G38550*, is strongly downregulated in *rdd* compared to the wild-type Col-0 plant [13,14]. The At5G38550p:GUS fusion transgene was also strongly downregulated in *rdd* compared to Col-0 in the current study (Figure 1). We previously proposed that this *rdd* downregulation is due to the promoter TEs [13]. However, in this study, the P-ΔTE1,2,3,4 GUS fusion construct, in which all four annotated TE sequences were deleted, still showed strong downregulation in *rdd* (Figure 4). This result suggested that the remaining part of the promoter sequence is responsible for *rdd* regulation of the *AT5G38550* gene. This is consistent with the location of the major differentially methylated region in the *AT5G38550* promoter, which is downstream of the last TE sequence before the transcription start site [13]. Further studies are needed to pinpoint the promoter sequence targeted by the DNA demethylases in *AT5G38550*. 

## 5. Conclusions

In this paper, we first demonstrated that the promoter fragments of DNA demethylase regulated defence-related genes in Arabidopsis with TE insertions contained the *cis*-regulatory information required for Fusarium-responsive and tissue-specific expression as well as for DNA demethylase regulation. Through successive promoter truncation, we demonstrated that deletion of a 201-bp short TE insertion in the promoter of the Jacalin lectin family protein gene, *AT5G38550*, abolished the root-specific expression pattern of the promoter. This study provides clear evidence that TE insertions play an important role in the expression of adjacent genes, and can provide *cis*-regulatory sequence elements for the neighbouring genes to gain specific spatiotemporal expression patterns.

## Supplementary Materials

The following are available online at http://www.mdpi.com/2073-4425/9/11/550/s1, Figure S1: The four genes selected for promoter study in this paper show strong down regulation in *rdd* based on both microarray (left; [1]) and mRNA deep sequencing (right; three biological replicates for each genotype) analyses of three week old plants; Figure S2: A typical trace file of bisulfite sequencing analysis of the chloroplast genome-encoded *psaA* gene, showing efficient bisulfite conversion. The original nucleotide sequence is shown above, with cytosines highlighted in blue. Note that all cytosines appear as thymines (red peak) in the sequencing trace of the PCR product. All bisulfite-treated samples used in this study showed a similar trace file, indicating efficient bisulfite conversion; Figure S3: Histochemical analysis of GUS expression in the T2 plants of the truncated At5G38550 promoter:GUS lines. Note that the root specificity of GUS expression is retained when the first two TEs were deleted, but lost after the deletion of the third TE sequence in the promoter fragment; Figure S4: The hAT TE sequence (At5TE55850) contains potential binding sites for AHL20 (AAAT) and ARR11 (GGATT)-binding motifs; Table S1: Oligonucleotide primers used in this study; Table S2: Number of primary transgenic lines obtained; Table S3: Transcription factor-binding motifs identified in AT5TE55850.
