# Supplementary material for: Root-Specific Expression of a Jacalin Lectin Family Protein Gene Requires a Transposable Element Sequence in the Promoter
Source: Genes (Basel). 2018 Nov 13;9(11):550. doi: 10.3390/genes9110550 (PMC6266147; doi:10.3390/genes9110550)
Supplement: Supplementary file 1 [file genes-09-00550-s001.zip › genes-381806-supplementary/genes-381806-Supplementary-Figures-final.pdf]

genes

Supplementary figures

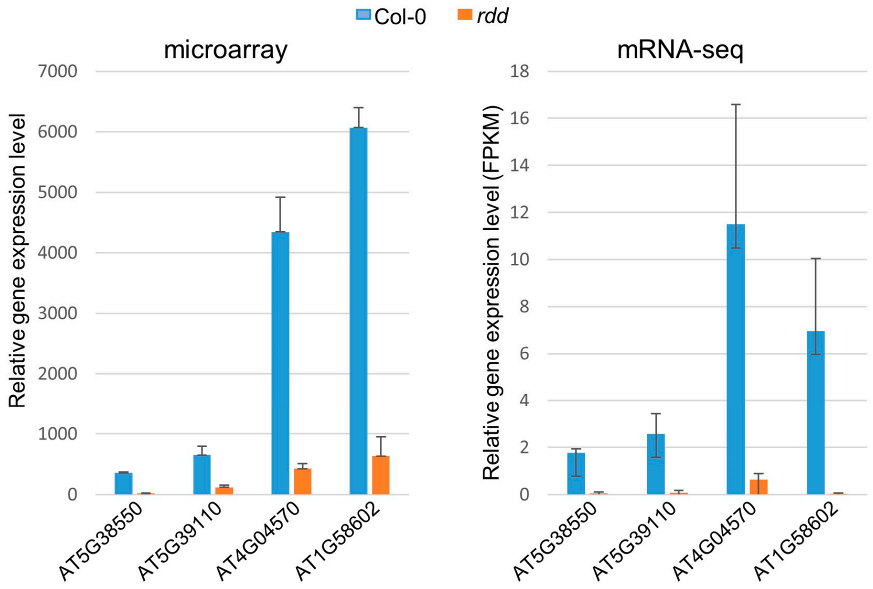

Figure S1. The four genes selected for promoter study in this paper show strong down regulation in *rdd* based on both microarray (left; [1]) and mRNA deep sequencing (right; three biological replicates for each genotype) analyses of three-week old plants.

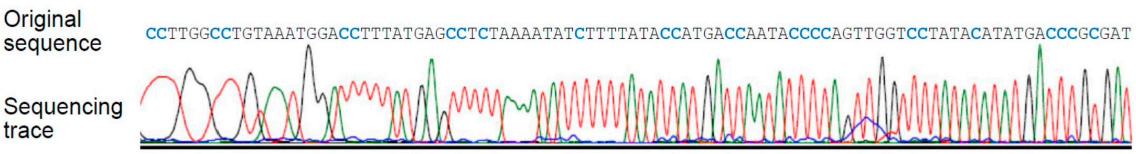

Figure S2. A typical trace file of bisulfite sequencing analysis of the chloroplast genome-encoded *psaA* gene, showing efficient bisulfite conversion. The original nucleotide sequence is shown above, with cytosines highlighted in blue. Note that all cytosines appear as thymines (red peak) in the sequencing trace of the PCR product. All bisulfite-treated samples used in this study showed a similar trace file, indicating efficient bisulfite conversion

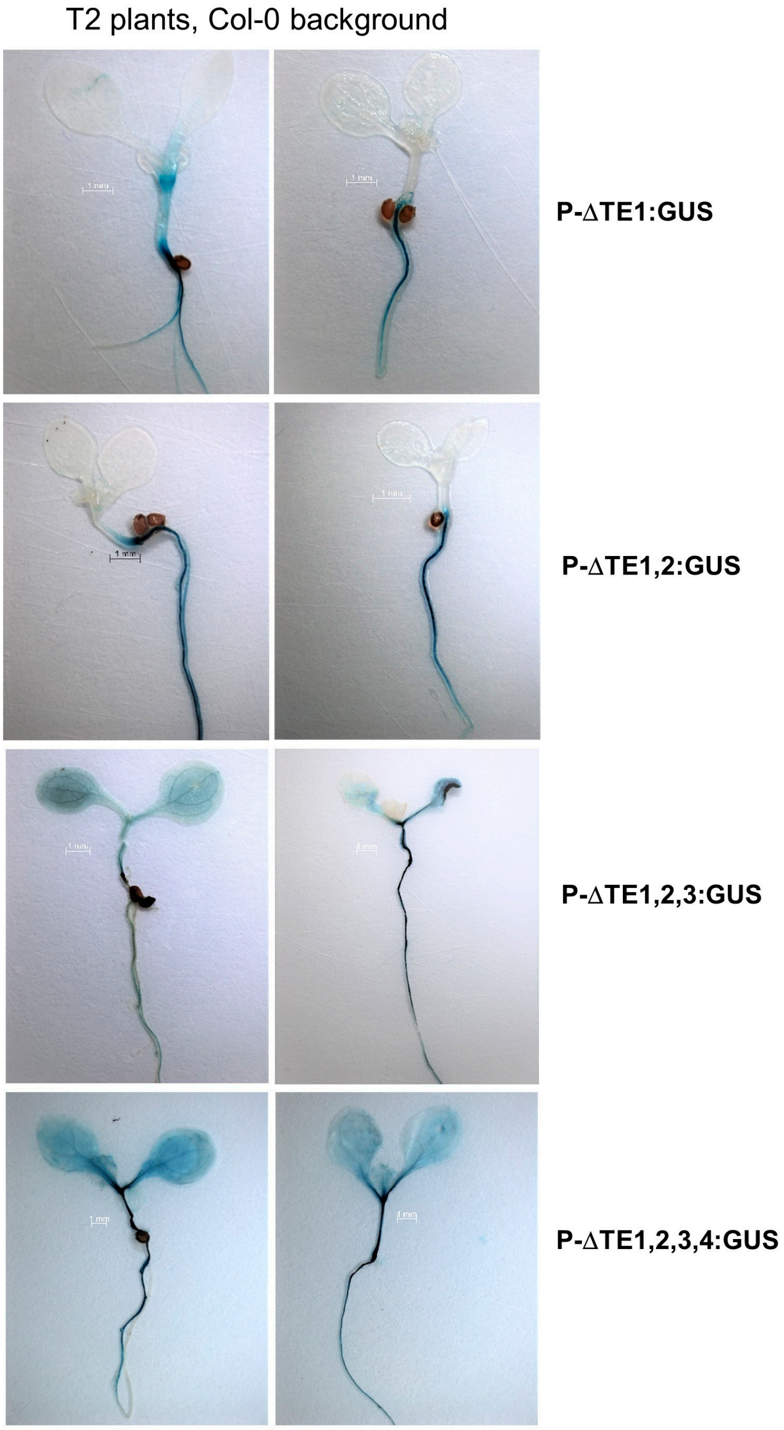

Figure S3. Histochemical analysis of GUS expression in the T2 plants of the truncated At5G38550 promoter:GUS lines. Note that the root specificity of GUS expression is retained when the first two TEs were deleted, but lost after the deletion of the third TE sequence in the promoter fragment.

Sequence of *AT5G38550* promoter :

ATCAGAGAGTTTTCCCTATCGTTTTCCTCGTTTATAATTGCATTTTTCATCGTTACTATT
CGTTAAATATTTTAATTTTTTTAATTAAAATATCTTTGCAGTTATGTCTTCTTACAAAGT
GTCTTTTCTCCATATTTATTTGTATTTTCCTTATCACAATTATTTCTTTCTAAAAATTAA
ATATTTTACAAATATTTGTGTCAATTTAATTGTTTATATCTTCTGAACTCTATATACTTT **AT5TE55860**
GTTTACCAAATATTCAATCTTCTGACAATTGTTTTTTTAAAGATGATTGATGGTCCAAGA
TTAAACCCGACCCGGACAGTGAACCCGACTACCTTCTCGATTATTGGGTCATCGGGTTAA
CCAGGTTGGACCATAAGTCAATTAATTAAAATTTATTTAATAAATTTAATATTAATAAGA
TTTTAGAGTTCTTATAAATTAAAAAATAAAAATAATCATGTTTATAAAATATAAAATCAG
ATTATGTTTAATAAAATATAAAATCATTTATACCAAAAAATAATAATAAAAAAAATAAAT
AATATATATATATATAATCAAACAATAATAATAAAGTGATTAAATTTTTTAAATCCAACT
AAACGTCTAATACCATAACAAATATAGTAGTAGATCACCAATGACGGAAATAAACTAGTA
AAACATCGTGAATTTACACAACTAATATCGGGTGTCTCACTCACCATTCGTTTCATTTTT
AAGGCGGCATTGTAAAGTCTTCGTCAAAATGTAAACCATAAAATGAGTTTACGAACTGAA
AAGATTTCTAAAAATGATTGAATAAAGTCTAAAATCTCATATTATATTCCAAAATAAAGA
GAATATTTTAAAACCCACACCCGACCGGTTCACCGGATCATAGGTTAATAGTGAGTTTTT
GTGGGTTTTTTTGATTTTGAACTTGTCGGGTTTTAGCACTAAACCTAAATCAGAAAAATG
TTTGGGTCACGGTTCAACCGGTCGGACCGAGTGTGAAAACACTGACCACTGGGTTTTTCT **AT5TE55855**
ATTTAAAAATAAGTTGAGAGTTTTAAAATGGATAAGTCTAAACTTGAGGGTTTTTTCATT
AAAATTTATTTTTCTAAAATAACAACAACAATTTATAATATTCATCATTTTGAGTTTGAT
TTATTGTTTGAGAAATATTGTATAAACAAATTTAAAATATTTTTCTTCATGAAATCGCGA
GAAAAGATATGTTTGAGTTTTATTAATAATAAAAATAATCAGATCATGTTTAACAAAATA
TAAAGTCTAACAATAATAAGAAAGTGATTAAAATTTATAAATCTAACTAAAACACGGTAA
AAAAATACTAAGATAGTAGGATAGTGAATACGAAGAACTTAAAAAAGTTTGAAATAATT
CAATAATGAACAGTGAATACACTAAAACTCTATCTTTTTGTCC**AAAATCAAATATATATA** **At5TE55850**
TTTTTAAACGAATACCCGACCAGTTTACCAGATCACAAGTTAGTGCCAAATTTTTGT**GGA**
TTTTTGCGCTTTTTAAATTGTCGGATATTAACAGTAAACTCAAACCCGATAAATATCTGT
AACACTGGTTAACCAGTA**GGATT**GGACGTGCAGATTTTTTTTTTCTTTTTTTTGAATGAAT
GACCAATGCTGAAGCTTTACGGTTTGGTTGCAAACCAGAGTTGCAGTCCATCTCTCATGG
AGGTTGAACCCATTTGAAGATATATATAGAAGCCGATTCCGAACTGTCCGGTCAATCACT **AT5TE55845**
TGAATTATTACCGATGCAGAGATGGGAAGCTCACCATGTCTTCTTCGATTTCTTTCCGTC
CAAATTGAATGGACTGTCGCTTTAAATATACGTAGCGTAAAATGAATTGATGCGTCTTCG
AGAACTGAGAAGTGGAGACAATACTTAGCAAAGCAGAGTAATCCAAAGTAAAATCTTGCA
TGAGAATTCGCTTGGCAAGATGTTCCCAGATTTGAGCCGAGAATGTGCAGCTGAAAAACA
AATGATCCCTTGTCTCAAATGGAACTTGACAAAACACACATGTT**GGATT**GAGATTCGATG
ACCAAGAGCACATTTGATCACATGTCACGTTGAAGTTGGTTTTATGTTTCTAAAAATAAT
GCCAAACAATAAATGCTCGAAACTGAAGTCCACCTACAAGTTTTACGAGTAGGTTTGTCA
TATCATAAAAAACAAAATCTACGCTGTCGATGAGATGCAATCATGCAAGGTCGTTTTAAA
ATAATCGTTTGTTTGTGGAAGACGTACTTTAGGTAGTCTTTGAATAATTATTTCACAACT
AGTTTATATTTTCACAAATTTAATATTTAATCTTGCAACCAGCAGCCTCATATATAACAA
TATAAATATACATTTTAGAAAAATGGCCAAAACTTGTATATATGAAAAAAGCCAAGTCCA
TCGCTA**AACTTACTGATCAGCATAGCAGTCTGTTCATCTTCAGAGGTCAGCTCTTTTCTC** **At5G38550 gene**
TTTCCCTCATAAGCATGCTTACTTCTTATTCGCACCAATGTTTTAACCTTAAATAAGATC
TTATCGAAATCTATTATTTATGTGAACGTAATGAGCATAGTTTTAGATATCCTCTTACAG
TAGTTCCGTT

Figure S4. The hAT TE sequence (At5TE55850) contains potential binding sites for AHL20 (AAAT) and ARR11 (GGATT)-binding motifs.

Reference

1. Le, T.N.; Schumann, U.; Smith, N.A.; Tiwari, S.; Au, P.C.; Zhu, Q.H.; Taylor, J.M.; Kazan, K.; Llewellyn, D.J.; Zhang, R.; et al. DNA demethylases target promoter transposable elements to positively regulate stress responsive genes in Arabidopsis. *Genome Biol.* **2014**, *15*, 458.
