# Supplementary material for: Root-Specific Expression of a Jacalin Lectin Family Protein Gene Requires a Transposable Element Sequence in the Promoter
Source: Genes (Basel). 2018 Nov 13;9(11):550. doi: 10.3390/genes9110550 (PMC6266147; doi:10.3390/genes9110550)
Supplement: Supplementary file 1 [file genes-09-00550-s001.zip › genes-381806-supplementary/genes-381806-Supplementary-Tables.pdf]

genes

MDPI

Supplementary Table 1. Oligonucleotide primers used in this study

**At5G38550 promoter fragment**	**Forward primer: CCTGCAGCTGGAAAGAATCTAAGTGCCTAG**<br>**Reverse primer: CGAGCTCCCTTGAACTGATGTTATCAATATTC**
At5G39110 promoter fragment	**Forward primer**: CCTGCAGCCATAAGCTATGGCTGTGGTTTTG<br>**Reverse primer**: CGAGCTCCCCTTTTCAAACAAAAGGTTATGCTAATCC
At4G04570 promoter fragment	**Forward primer**: CCTGCAGCCCATCTCCATAACTCTCTCG<br>**Reverse primer**: CGAGCTCCCGGTTTTGTTTGTTAAAGATAACAACTTC
At1G58602 promoter fragment	**Forward primer**: CCTGCAGCCTCACCACACAAACTCTATATCAC<br>**Reverse primer**: CGAGCTCCCTTTTGAATCTTGATCGCGCC
At5G38550 truncated promoter fragments	**Reverse primer for all** (same as above for full-length promoter):<br>CGAGCTCCCTTGAACTGATGTTATCAATATTC<br>**Forward primer for P-ΔTE1,2,3,4**:CTGCAGATGTCACGTTGAAGTTGGTTTTATG<br>**Forward primer for P-ΔTE1,2,3**: CTGCAGGAATGAATGACCAATGCTGAAGC<br>**Forward primer for P-ΔTE1,2**: CTGCAGGAACAGTGAATACACTAAAACTC<br>**Forward primer for P-ΔTE1,2**:CTGCAGACCACTGGGTTTTTCTATTTAAAAATAAG
Bisulfite primers for AT5G38550p:GUS	**Bisulfite region 1**:<br>Primary PCR<br>**AT5G38550-Bi-F1** (GBis-F1): TTTTTAAGGYGGYATTGTAAAG<br>**AT5G38550-Bi-R1** (GBis-R1): TAAATTTRTTTATACAATATTTCTC<br>Nested PCR<br>**AT5G38550-Bi-F1** (GBis-F1: TTTTTAAGGYGGYATTGTAAAG<br>**AT5G38550-Bi-R2** (GBis-R2: TCTCAAACAATAAATCAAACTC<br><br>**Bisultife region 2**:<br>Primary PCR<br>**AT5G38550-Bi-F2** (GBis-F2): TTAYYGATGYAGAGATGGGAAG<br>**AT5G38550-Bi-R3** (GBis-R3): TRTRAAATAATTATTCAAARACTACC<br>Nested PCR<br>**AT5G38550-Bi-F2** (GBis-F2): TTAYYGATGYAGAGATGGGAAG<br>**AT5G38550-Bi-R4** (GBIS-R4): AARTACRTCTTCCACAAACAAAC<br><br>**Bisulfite region 3**:<br>Primary PCR<br>**AT5G38550-Bi-F3** (023-Bi-F2): GYAGTYTGTTYATYTTYAGAGG<br>**GUS-Bi-R1** (018-Bi-R1): CACCAACRCTRATCAATTCCAC<br>Nested PCR<br>**AT5G38550-Bi-F3** (023-Bi-F2): GYAGTYTGTTYATYTTYAGAGG<br>**GUS-Bi-R2** (018-Bi-R2; Pro:GUS junction): CRATRAATTCRARCTCRRTACCC
Bisulfite primers for AT4G04570p:GUS	**Bisulfite region 1:**<br>Primary PCR<br>**LB-Bis-F1** (018-BI-F1): GTGGYAGGATATATTGTGGTG<br>**AT4G04570p-Bi-R1** (025-BI-R1): CRCRTCTTTRTTAACTTCCAAAC<br>Nested PCR<br>**LB-Bis-F1** (018-Bi-F2): TGTAYTGAATTYTYATGTTTG<br>**AT4G04570p-Bi-R2** (025-Bi-R2): CAACAACRARCTRATAAACATC<br>**AT4G04570p-Bi-R3** (025-Bi-R3): ARAAARARACACAACTCTTTAC<br><br>**Bisulfite region 2:**<br>Primary PCR<br>**AT4G04570p-Bi-F1** (025-BI-F1): AGAGGAAYYATTGTAAAATAG<br>**GUS-Bi-R1** (018-Bi-R1): CACCAACRCTRATCAATTCCAC<br>Nested PCR<br>**AT4G04570p-Bi-F2** (025-Bi-F2): GTYAATGGTYGTATGTGAATGG<br>**AT4G04570p-Bi-F3** (025-Bi-F3): YATTGAAAGATAAYTTGTG<br>**GUS-Bi-R2** (018-Bi-R2; Pro:GUS junction): CRATRAATTCRARCTCRRTACCC

Supplementary Table 2. Number of primary transgenic lines obtained

Constructs	No lines in Col-0	No. lines in *rdd*
AT5G38550p:GUS	23	24
AT5G39110p:GUS	15	12
AT4G04570p:GUS	25	28
AT1G58602:GUS	16	14
P-ΔTE1:GUS	19	10
P-ΔTE1,2:GUS	16	14
P-ΔTE1,2,3:GUS	14	11
P-ΔTE1,2,3,4:GUS	18	22

Supplementary Table 3. Transcription factor-binding motifs identified in At5GTE55850

Cis-element	Family	Time of occurrence	Expression pattern of TF in AtGenExpress database
AHL12	AT-hook	9	Shoot apex and flowers
AHL20	AT-hook	12	**Root-specific**
AHL25	AT-hook	5	unrecorded
ARR11	GARP/ARR-B	5	**Root-preferential**
DOF2	C2C2 (Zn)	2	Constitutive
DOF5.7	C2C2 (Zn)	3	unrecorded
GT1	Trihelix	4	Constitutive
KAN1	GARP	2	Constitutive
RVE1	MYB	5	Constitutive
ZmHOX2a	HD-HOX	2	unrecorded
